# P-119. Thrombocytopenia and Clinical Correlates in Infectious Encephalitis

**DOI:** 10.1093/ofid/ofaf695.347

**Published:** 2026-01-11

**Authors:** Cassidy M Edwards, Rodrigo Hasbun, Arun Venkatesan, Ralph Habis, John Probasco

**Affiliations:** The University of Texas Health Sciences Center in Houston, Houston, TX; UT Health Mc Govern Medical School, Houston, Texas; Johns Hopkins University School of Medicine, Baltimore, Maryland; Johns Hopkins University School of Medicine, Baltimore, Maryland; Johns Hopkins University School of Medicine, Baltimore, Maryland

## Abstract

**Background:**

Encephalitis is characterized by inflammation of the brain and is most commonly caused either by infectious or autoimmune etiologies. Encephalitis is associated with significant neurological morbidity mortality. Several studies, including our prior study investigating risk factors for mortality in encephalitis^2^, have identified thrombocytopenia as an independent predictor for mortality.^1-4^ This study aimed to investigate the clinical and prognostic significance of thrombocytopenia in a large cohort of adults with encephalitis.

**Figure:**
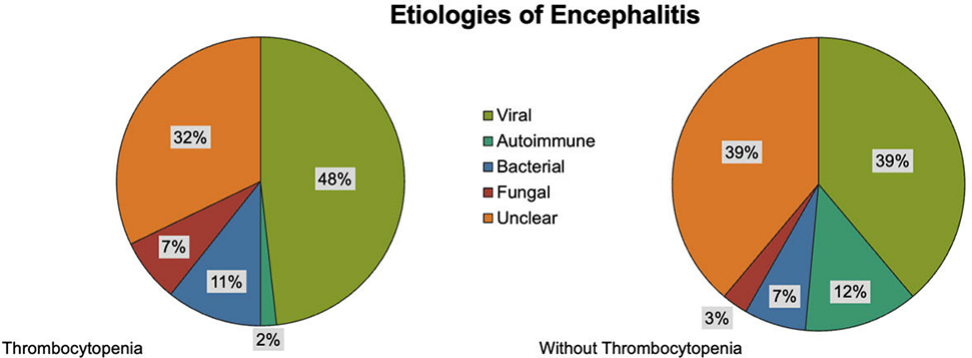

**Figure:**
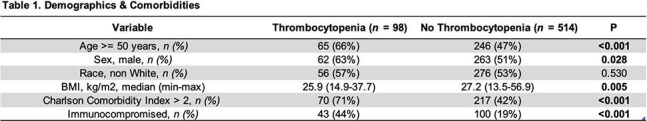

**Methods:**

We conducted a retrospective review of adults (≥ 18 years) with encephalitis-related ICD-9 diagnoses from two hospital systems in Houston (2005–2023) and Baltimore (2002–2022). A total of 612 patients (327 from Texas, 240 from Maryland) met International Encephalitis Consortium and/or Graus criteria and were included. Patients with unclear thrombocytopenia status were excluded. This retrospective review study was approved by the Institutional Review Boards of The University of Texas Health Science Center at Houston and Johns Hopkins Hospital.

**Figure:**
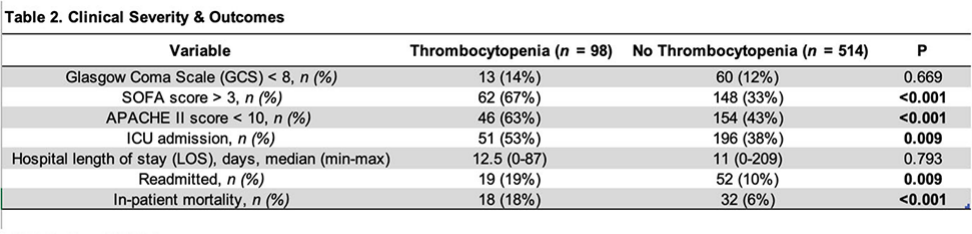

**Figure:**
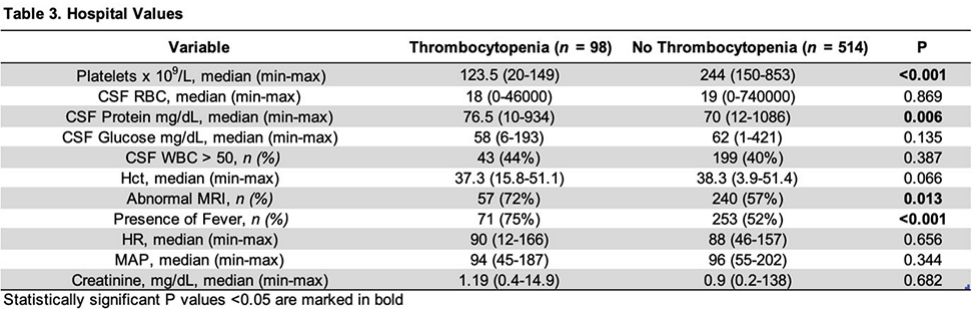

**Results:**

Among 612 encephalitis patients analyzed to date, 16% presented with thrombocytopenia. In both groups, the most common etiology of encephalitis was viral (see Figure 1). Patients with thrombocytopenia were more likely be older, have more comorbidities, be immunosuppressed and have lower BMI (p < 0.05) (see Table 1). Furthermore, patients with thrombocytopenia were also sicker (higher APACHE 2 scores, higher SOFA scores, higher ICU admission rates, higher CSF protein, higher proportion of fever and abnormal MRIs of the brain) (P < 0.05) (see Tables 2 and 3). Finally, encephalitis patients with thrombocytopenia had higher readmission rates (19% vs 10%, P = 0.009) and higher in-hospital mortality (18% vs 6%, p = < 0.001).

**Conclusion:**

In adults with encephalitis, thrombocytopenia is seen more commonly in older patients with comorbidities who are sicker on initial presentation and is significantly associated with higher rates of readmission and inpatient mortality. Further investigation in the causes of thrombocytopenia in this population could elucidate potential mechanisms of this important prognostic factor in encephalitis.

**Disclosures:**

Rodrigo Hasbun, MD MPH FIDSA, Biomeriaux: Grant/Research Support|Biomeriaux: research funding and personal fees to help with Monte Carlo simulation studies evaluating impact of cost on adults and children John Probasco, MD, Genentech: Site investigator

